# Clinicopathological and prognostic significance of metastasis-associated in colon cancer-1 (MACC1) overexpression in colorectal cancer: a meta-analysis

**DOI:** 10.18632/oncotarget.11287

**Published:** 2016-08-14

**Authors:** Yang Zhao, Cong Dai, Meng Wang, Huafeng Kang, Shuai Lin, Pengtao Yang, Xinghan Liu, Kang Liu, Peng Xu, Yi Zheng, Shanli Li, Zhijun Dai

**Affiliations:** ^1^ Department of Oncology, Second Affiliated Hospital of Xi'an Jiaotong University, Xi'an, China

**Keywords:** MACC1, colorectal cancer, prognosis, meta-analysis

## Abstract

Metastasis-associated in colon cancer-1 (MACC1) has been reported to be overexpressed in diverse human malignancies, and the increasing amount of evidences suggest that its overexpression is associated with the development and progression of many human tumors. However, the prognostic and clinicopathological value of MACC1 in colorectal cancer remains inconclusive. Therefore, we conducted this meta-analysis to investigate the effect of MACC1 overexpression on clinicopathological features and survival outcomes in colorectal cancer. PubMed, CNKI, and Wanfang databases were searched for relevant articles published update to December 2015. Correlation of MACC1 expression level with overall survival (OS), disease-free survival (DFS), and clinicopathological features were analyzed. In this meta-analysis, fifteen studies with a total of 2,161 colorectal cancer patients were included. Our results showed that MACC1 overexpression was significantly associated with poorer OS and DFS. Moreover, MACC1 overexpression was significantly associated with gender, localization, TNM stage, T stage, and N stage. Together, our meta-analysis showed that MACC1 overexpression was significantly associated with poor survival rates, regional invasion and lymph-node metastasis. MACC1 expression level can serve as a novel prognostic factor in colorectal cancer patients.

## INTRODUCTION

Colorectal cancer (CRC), which is the third most common cause of cancer and the third most common cause of cancer death after lung cancer, prostate cancer in men, and breast cancer in women, is a worldwide disease [1]. Every year, more than 1.2 million patients are diagnosed with colorectal cancer, from which more than 600,000 die [2]. Metastases arising from residual colorectal tumors is the major source of cancer-related deaths, approximately 90% of patient deaths. 5-year survival rates for patients with early stage, regional lymph node metastasis, and metastatic diseases are ≥ 90%, 65%, and ≤ 10%, respectively [3]. Therefore, early discovery of tumor occurrence and metastasis is critical to modify therapeutic strategies and improve patient prognosis. However, it is not determined to accurately predict the development of metastasis of CRC on the basis of the current clinicohistopathological classifications and molecular biomarkers. Therefore, the predictive biomarker for the early and accurate identification of high risk for metastasis in patients with CRC is ought to help to improve the clinical individual therapy.

Metastasis-associated in colon cancer-1 (MACC1) gene located at 7p21.1 was identified by a genome-wide search for a set of differently expressed genes in primary and metastatic colon cancer [4]. In the cultured cells, MACC1 was able to promote proliferation, migration, and dissemination, and to regulate gene transcription *via* the hepatocyte growth factor (HGF) /mesenchymal-epithelial transition factor signaling pathways [4]. Subsequent clinical investigations showed that MACC1 might be useful in the prognostic classification of colorectal cancer patients and was a promising new target for intervention in metastasis [4–8]. However, the relationship between MACC1 expression level and prognostic / clinicopathological outcomes in CRC patients remains controversial. Therefore, we conducted this meta-analysis.

## RESULTS

### Search results and study characteristics

**Figure 1:**
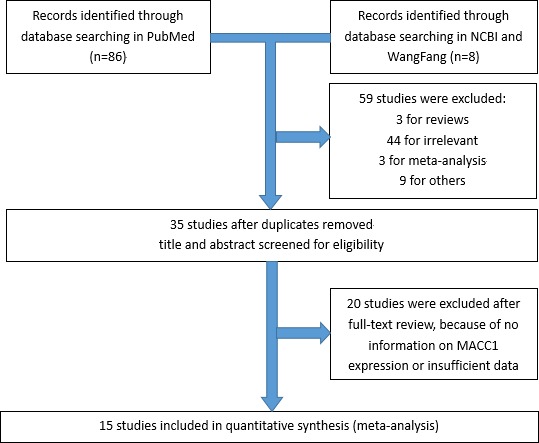
Flow chart of the selection of the studies in the meta-analysis

A total of thirty-five articles were initially identified using the search criteria delineated above. As shown in Figure 1, twenty were excluded owing to irrelevance to the analysis or insufficient primary outcome. There were fifteen studies included in this meta-analysis [4, 5, 8–20].

The characteristics of the fifteen studies are summarized in Table 1. Of fifteen publications, eleven assessed the relationship between MACC1 expression and CRC clinicopathological features. Ten studies evaluated the association of MACC1 expression and prognosis in patients with CRC (four evaluated OS and six evaluated DFS). A total of 2,161 patients from China, Greece, Germany, America, Italy, and Japan were enrolled with sample number ranging from 60 to 323. To observe MACC1 status in CRC, immunohistochemistry (IHC) was used in eight studies and reverse transcription-polymerase chain reaction (RT-PCR) was used in seven. The median positive rate of MACC1 was 60.2% (30.0%-86.7%). Meanwhile, the follow-up times ranged from 20 to 56.2 months (Table 1). The study quality was assessed using the Newcastle-Ottawa quality assessment scale, generating scores ranging from 6 to 8 with a mean of 6.7.

**Table 1:** Main characteristics of all studies included in the meta-analysis

First author	Year	Patient source	Number of patient	Stage	Method	MACC1 expression (%)	Median(range) Follow-up(month)	Outcome	Multivariate/Univariate	HR(95%Cl)	Scores of study
Schmid^20^	2015	Germany	60	I-III	RT-PCR	51.7	NR	DFS	Univariate	1.71(0.41-7.23)	6
Koelzer^10^	2015	Greece	187	I-IV	IHC	58.3	NR	NR	NR	NR	6
Katharina^11^	2015	Germany	99	I-III	RT-PCR	35.4	56.0	DFS	Multivariate	6.09(2.50-14.85)	8
Xu^9^	2015	China	96	I-IV	IHC	75	13(4-21)	NR	NR	NR	6
Ge^14^	2015	China	96	II-IV	IHC	53.1	30.27	DFS	Multivariate	2.11(1.32-3.38)	7
Yamamoto^12^	2014	Japan	174	I-IV	RT-PCR	82.2	49.2	DFS	Multivariate	2.27(1.01-9,71)	8
Zhen^13^	2014	China	323	I-IV	RT-PCR	52.3	NR	OS	Multivariate	1.410(0.737-2.699)	7
Ren^17^	2013	America	93	I-II	IHC	73.1	20(2-62)	NR	NR	NR	6
Liu^15^	2013	China	128	I-IV	IHC	65.0	NR	NR	NR	NR	6
Kang^16^	2013	China	317	I-IV	IHC	61.8	NR	OS	Univariate	2.67(1.57-4.55)	6
Isella^8^	2013	Italy	64	NR	RT-PCR	79.7	33	DFS	Multivariate	7.274(1.658-31.91)	7
Zhang^18^	2012	China	90	I-IV	IHC	86.7	47.1	OS	Univariate	0.46(0.06-3.50)	8
Zhou^19^	2012	China	80	I-III	IHC	41.3	NR	NR	NR	NR	6
Stein^5^	2012	Germany	294	I-IV	RT-PCR	49	28	OS	Univariate	4.89(1.90-12.59)	7
Stein^4^	2009	Germany	60	I-III	RT-PCR	30	47.2	DFS	Univariate	3.340(1.820-6.130)	6

### Quantitative synthesis

#### MACC1 expression and clinicopathological parameters

To investigate the association between MACC1 expression and clinicopathological features, we conducted the meta-analysis. Accordingly, our results showed that increased MACC1 expression was significantly correlated to gender (OR = 0.804, 95% CI = 0.654-0.988, fixed-effect model), localization (OR = 2.669, 95% CI = 1.586-4.492, fixed-effect model), TNM stage (OR = 1.976, 95% CI = 1.495-2.612, fixed-effect model), T stage (OR = 2.002, 95% CI = 1.548-2.589, fixed-effect model), and N stage (OR = 3.182, 95% CI = 1.472-6.877, random-effect model). On the contrary, MACC1 overexpression was not found to be associated with age (OR = 1.200, 95% CI = 0.834-1.726, random-effect model), tumor size (OR = 1.475, 95% CI = 0.798-2.728, random-effect model), tumor grade (OR = 1.329, 95% CI = 0.851-2.076, fixed-effect model), or distant metastasis (OR = 1.925, 95% CI = 0.761-4.870, random-effect model). These results suggested that CRC with overexpressed MACC1 exhibited aggressive biological behaviors (Table 2).

**Table 2:** Meta-analysis for the association of increased MACC1 expression and clinicopathological features of CRC patients

Clinicopathological features	No. of studies	No. of patients	Model	OR(95% CI)	*P*-Value	Heterogeneity		
						χ2	I^2^ (%)	*P*-Value
Age(younger *vs*. older)	10	1500	Random	1.200(0.834,1.726)	0.326	21.47	58.1	0.011
Size(smaller *vs*. bigger)	7	994	Random	1.475(0.798,2.728)	0.215	22.53	73.4	0.001
Gender(male *vs*. female)	11	1682	Fixed	0.804(0.654,0.988)	0.038	11.09	9.8	0.351
Localization(Colon *vs*. Rectum)	3	319	Fixed	2.669(1.586,4.492)	0.000	2.71	26.1	0.258
Tumor grade(G1-2 *vs*. G3-4)	3	382	Fixed	1.329(0.851,2.076)	0.211	1.02	0.0	0.599
TNM stage(I-II *vs*. III-IV)	6	891	Fixed	1.976(1.495,2.612)	0.000	6.01	16.8	0.305
T stage(Tis-T2 *vs*. T3-T4)	8	1306	Fixed	2.002(1.548,2.589)	0.000	13.61	48.6	0.059
N stage(N0 *vs*. N1-N2)	6	1034	Random	3.182(1.472,6.877)	0.003	33.09	84.9	0.000
Distant metastasis(M0 *vs*. M1)	6	1128	Random	1.925(0.761,4.870)	0.167	17.44	71.3	0.004

#### MACC1 expression and OS in colorectal cancer

**Figure 2:**
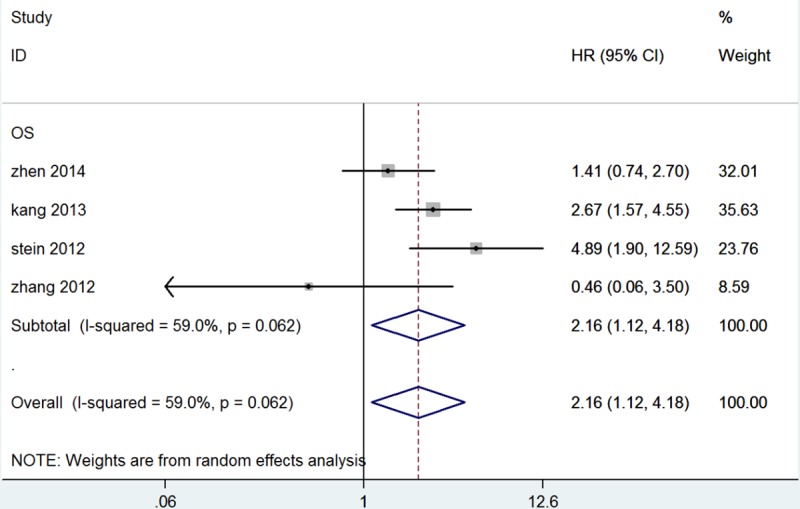
Forest plot of hazard ratio for the association of MACC1 overexpression and overall survival

Overall, four studies, including 1,024 patients, had a relationship between OS and MACC1 expression level. Heterogeneity among studies was statistically significant (*P* = 0.062, I^2^ = 59.0%), so a random-effects model was used. The pooled HR for OS showed overexpression of MACC1 was significantly associated with reduced OS in CRC (HR = 2.16, 95% CI = 1.12-4.18, *P* = 0.022, Table 3 and Figure 2). We also performed subgroup analysis for ethnicity, method, and HR estimate. Subgroup analysis for ethnicity showed an insignificant association in Asian studies (HR = 1.718, 95% CI = 0.863-3.417, *P* = 0.123). In the subgroup analysis for method of detection, the results suggested that the detection method, either RT-PCR or IHC, of MACC1 expression did not significantly influence outcomes (RT-PCR: HR = 2.499, 95% CI = 0.742-8.421, *P* = 0.139; IHC: HR = 1.474, 95% CI = 0.289-7.524, *P* = 0.641). For HR estimation, subgroup analysis suggested that the overall HR estimate for OS with univariate analysis was 2.598 (95% CI = 1.106-6.102, *P* = 0.028) (Table 3).

**Table 3:** Main meta-analysis results

Analysis	No. of studies	No. of patients	Model	HR(95% CI)	*P*-Value	Heterogeneity	
						I^2^ (%)	*P*-Value
**OS**	4	1024	Random	2.16(1.12,4.18)	0.022	59.0	0.062
**Ethnicity**							
Asian	3	730	Random	1.718(0.863,3.417)	0.123	52.8	0.120
Non-Asian	1	294	-	4.890(1.900,12.588)	0.001	-	-
**Method**							
IHC	2	407	Random	1.474(0.289,7.524)	0.641	62.8	0.101
RT-PCR	2	617	Random	2.499(0.742,8.421)	0.139	77.9	0.034
**HR estimate**							
Multivariate analysis	1	323	-	1.410(0.737,2.698)	0.299	-	-
Univariate analysis	3	701	Random	2.598(1.106,6.102)	0.028	54.5	0.111
**DFS**	6	553	Fixed	2.86(2.09,3.91)	0.000	26.6	0.235
**Ethnicity**							
Asian	2	270	Fixed	2.133(1.382,3.292)	0.001	0.0	0.907
Non-Asian	4	283	Fixed	3.921(2.497,6.155)	0.000	5.1	0.367
**Method**							
IHC	1	96	-	2.110(1.319,3.376)	0.002	-	-
RT-PCR	5	457	Fixed	3.638(2.392,5.531)	0.000	0.0	0.415
**HR estimate**							
Multivariate analysis	4	433	Random	3.346(1.756,6.374)	0.000	50.4	0.109
Univariate analysis	2	120	Fixed	3.017(1.725,5.278)	0.000	0.0	0.400

OS, Overall survival; DFS, Disease-free survival

#### MACC1 expression and DFS in colorectal cancer

**Figure 3:**
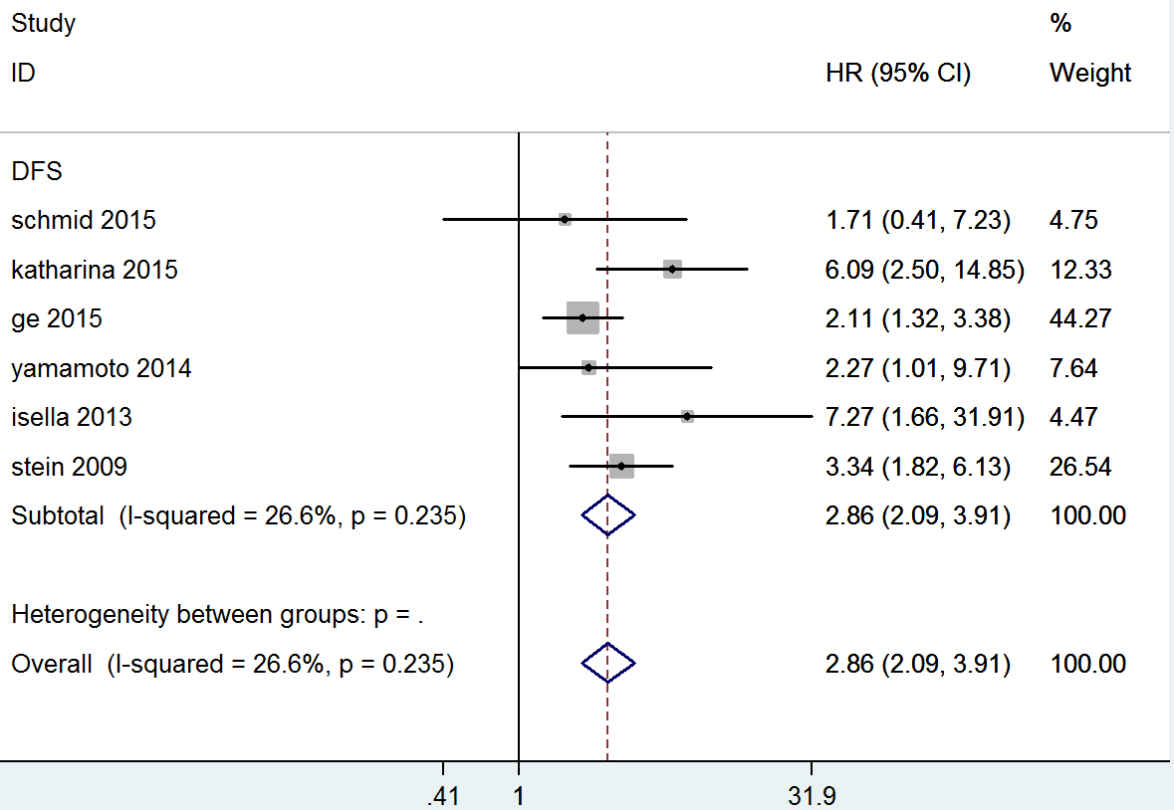
Forest plot of hazard ratio for the association of MACC1 overexpression and disease-free survival

Six studies comprising a total 553 patients provided results regarding to DFS. There was no significant heterogeneity (*P* = 0.235, I 2 = 26.6%) among them, so a fixed-effect model was used to calculate the pooled HR and 95% CI. Our results showed that increased MACC1 expression was significantly associated with poorer DFS (HR = 2.86, 95% CI = 2.09-3.91, *P* = 0.000), indicating that increased MACC1 expression was an indicator of disease recurrence in CRC patients (Table 3 and Figure 3). Meanwhile, a tight association was also observed between MACC1 expression and CRC patient prognosis across various geographic regions; the pooled HR was 2.133 (95% CI = 1.382-3.292, *P* = 0.001) from Asia, and HR was characterized as 3.921 (95% CI = 2.497-6.155, *P* = 0.000) from non-Asian countries. Subgroup analysis by method and HR estimation suggested a significant association in RT-PCR (HR = 3.638, 95% CI = 2.392-5.531, *P* = 0.000), multivariate analysis (HR = 3.346, 95% CI = 1.756-6.374, *P* = 0.000) and Univariate analysis (HR = 3.017, 95% CI = 1.725-5.278, *P* = 0.000) (Table 3).

#### Publication bias

**Figure 4:**
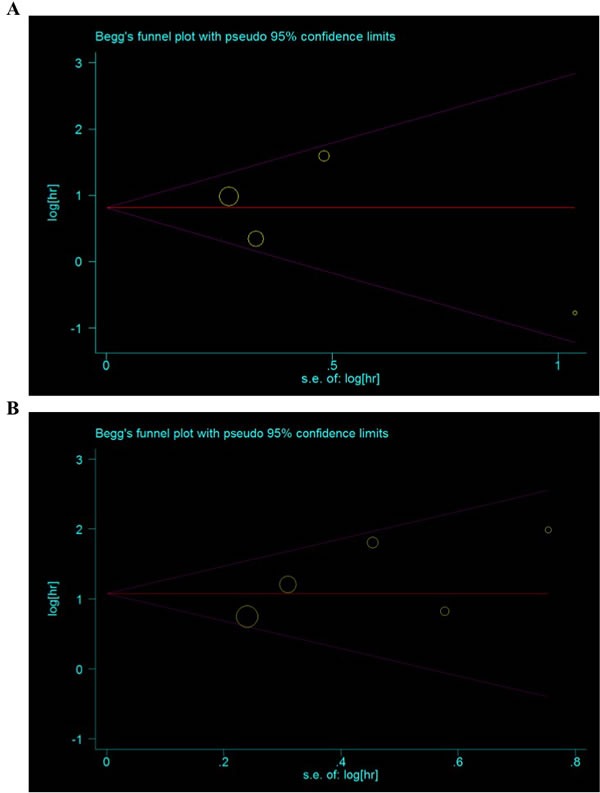
Funnel plots of publication bias in this meta-analysis **A.** Overall survival; **B.** Disease-free survival.

In this meta-analysis, both Begg's and Egger's tests were performed to assess if any publication bias existed in the published literature. No publication bias was observed among studies with OS (*P* = 1.000, 0.679) and DFS (*P* = 0.707, 0.433). The Begg's plots for the effect of MACC1 expression level on prognosis were shown in Figures 4A and 4B.

## DISCUSSION

Identification of a novel predictive and prognostic marker to guide clinical therapy for patients with CRC is currently of special interest. Many molecular markers, such as TP53 [21], KRAS, and BRAF [22], have been investigated over the past few years. Notwithstandingly, they are not routinely used in clinical practice due to their accuracy and stability to predict the prognosis of CRC. In recent years, some studies have shown the potential clinical value of MACC1 to serve as a prognostic indicator and a potential novel target for treatment in patients with CRC.

The first study investigating the relationship between MACC1 and survival patients with cancers was reported by Stein et al. Their results suggested that MACC1 expression level acted as an independent prognostic indicator of tumor metastasis and disease-free survival (DFS) [4]. Recently, a number of studies have been carried out to investigate the correlation of MACC1 expression to the survival and prognosis of CRC patients, but results have been inconsistent. Meta-analysis have been performed to resolve controversial results for identification of prognostic indicators in patients with malignant diseases, and more recently, this approach has been applied widely and successfully. Therefore, we conducted a meta-analysis of the evidence obtained from all published MACC1/CRC studies in order to provide a quantitative reassessment of this association.

The present study was the meta-analysis of published data regarding to the relationship between MACC1 and disease prognosis in patients with CRC. We observed a relationship between MACC1 overexpression, poorer overall survival, and disease-free survival, and among subgroups defined by study region. Furthermore, we observed an association between MACC1 overexpression and several clinicopathological parameters (patient age, tumor size, patient gender, cancer localization, tumor grade, TNM stage, nodal status, tumor depth, and distant metastases). This meta-analysis engenders several important implications. First of all, we observed a positive relationship between MACC1 overexpression and several clinicopathological parameters. Females with colon cancer displayed a poorer prognosis as compared to males with rectal cancer. In addition, the pooled results of TNM stage, nodal status, and tumor depth suggested that increased MACC1 expression promoted regional invasion and lymph-node metastasis, thus leading to poorer CRC prognosis. However, we can also see from the results that increased MACC1 expression was not correlated with distant metastasis. This lead us to conclude that MACC1 overexpression might be associated with CRC progression, but this has to be proved by research using sufficiently large sample sizes. Pooled statistical data, however, did show that overexpression of MACC1 was associated with worse survival outcomes, including OS and DFS. These results indicated that MACC1 might serve as a new parameter for predicting outcomes and a potential novel target for treatment in CRC patients. Finally, when extended to subgroup analysis of analysis method of MACC1, The same conclusion is found between RT-PCR and IHC. Similarly, from the point of ethnicity and HR estimate, we also have come to the same conclusion, although this may result from too-small sample sizes, causing a potential type I error. Therefore, it is necessary to perform better-designed studies using huge sample to confirm or to refute our findings.

In our meta-analysis, we found significant heterogeneity across the included studies. The I^2^ values for OS and DFS were 59.0% and 26.6%, respectively. Although we used random-effect models to pool the OS data, the models did not identify the source of heterogeneity. In addition, a random-effect model can reduce the effect of large-sample studies of good quality. Although the exact sources of heterogeneity were not well-clarified, there were several possible reasons for this heterogeneity, such as the detection method for measuring MACC1 expression levels, the small number of included studies, the differences in TNM stage, and the statistical approach for extrapolating HRs. However, since we found no publication bias, statistical results seemed to be robust and convincing. The data on MACC1 expression and CRC prognosis is promising, but strongly supported the further clinical study to uncover the potential and value of MACC1 to function as a prognoticator.

In conclusion, we found that MACC1 expression indicated poor survival outcomes and regional invasion and lymph-node metastasis. Therefore, we believe that MACC1 can serve as a prognostic indicator and a potential novel target for treatment in CRC patients. Larger and more well-designed studies are required to clarify the prognostic significance of MACC1 expression in CRC patients.

## MATERIALS AND METHODS

This meta-analysis was conducted in accordance with the Preferred Reporting Items for Systematic Reviews and Meta-Analyses (PRISMA) guidelines.

### Identification and selection of studies

Studies were identified by searching PubMed, CNKI, and WanFang databases covering all papers published update to December 2015. The following search strategy was used: “colon cancer or colon carcinoma or rectum cancer or rectum carcinoma or colorectal cancer or colorectal carcinoma” and “MACC1 or Metastasis-associated in colon cancer-1”. No language restrictions were applied. All eligible studies were retrieved, and their references were cross-searched to triage additional suitable studies. Once publications were found with overlapping data published by the same investigator, only the most complete report was included. Disagreements were resolved by iteration, discussion, and consensus between the two authors.

Studies were included if they fulfilled the following criteria: (a) reporting explicit methods for the detection of MACC1 expression in CRC; (b) the endpoints were to evaluate the relationship of MACC1 expression in CRC patients with OS, DFS, and a series of clinicopathological parameters; and (c) provided a hazard ratio (HR) or odds ratio (OR) with the corresponding confidence interval (CI) or sufficient data to calculate them. Articles were excluded from the analysis following criteria: (a) letters, case reports, reviews, and conference abstracts without original data; (b) duplicates of previous publications; (c) articles without key information such as Kaplan-Meier curves, hazard ratios (HRs) with the 95% confidence intervals (CIs), or clinicopathological features.

### Data extraction

Two independent reviewers extracted the details of included studies with a standardized form. The following information was recorded: first author's surname, year of publication, number of patient, patient source, tumor stage, MACC1 assessment method, MACC1 expression, follow-up time, prognostic outcomes, analytical method, and HR with its 95 % CI. If the above-mentioned data was not reported, items should be treated as “NR (not reported)”.

### Methodological quality of the studies

The Newcastle-Ottawa Scale was used to assess the quality of each study [23]. The NOS criteria is scored based on three aspects: (1) subject selection, (2) comparability of subject, (3) clinical outcome. NOS scores range from 0 to 9, and a score ≥ 6 indicates a high quality. Two investigators independently assessed the quality of the 9 included studies, and the discrepancies were solved by consensus.

### Statistical methods

Included studies were divided into three groups: OS, DFS, and clinicopathological parameters. MACC1 was considered as ‘high’ or ‘low’ expression according to the cut-off values provided by the authors in each publication, because of variation on the definition for the ‘high’ or ‘low’ expression of MACC1 among studies. Hazard ratios (HRs) and 95% CIs were combined to measure the effective value. For these HRs that were given explicitly in the published studies, we used crude ones. If not, we calculated the values from the Kaplan-Meier survival curve or the available data using methods reported by Parmar et al [24]. Data from the Kaplan-Meier survival curves were read using Engauge Digitizer version 4.1. A combined HR/OR > 1 indicated a poor outcome for MACC1 overexpression, while HR/OR < 1 indicated a favorable outcome for MACC1 overexpression. Heterogeneity among the studies was determined by chi-square test and Q test. If heterogeneity was significant (*P* < 0.1 or I^2^ > 50%), the DerSimonian and Laird random-effects model were used [25]. Otherwise, a fixed-effects model of Mantel-Haenszel was applied in the absence of between-study heterogeneity [26]. Both Egger's and Begg's tests were used to examine publication bias [27, 28]. All *P* values were two-sided, and *P* < 0.05 was considered as statistically significant. Statistical calculations were performed using STATA 12.0.

## SUPPLEMENTARY MATERIALS



## Abbreviations

MACC1: metastasis-associated in colon cancer-1
CRC: colorectal cancer
HR: hazard ratios
OR: odds ratio
CI: confidence interval
OS: overall survival
DFS: disease-free survival
IHC: immunohistochemistry
RT-PCR: reverse transcription-polymerase chain reaction
